# Robotic-enhanced hybrid ablation for inappropriate sinus tachycardia: a world-first approach

**DOI:** 10.1093/icvts/ivae184

**Published:** 2024-11-14

**Authors:** Zain Khalpey, Ujjawal Kumar, Alyssa Abraham, Yoaav Krauthammer

**Affiliations:** Department of Cardiac Surgery, HonorHealth, Scottsdale, AZ 85258, USA; Khalpey AI Lab, Applied & Translational AI Research Institute (ATARI), Scottsdale, AZ 85258, USA; Department of Cardiac Surgery, HonorHealth, Scottsdale, AZ 85258, USA; Khalpey AI Lab, Applied & Translational AI Research Institute (ATARI), Scottsdale, AZ 85258, USA; School of Clinical Medicine, University of Cambridge, Cambridge, CB2 0SP, UK; Khalpey AI Lab, Applied & Translational AI Research Institute (ATARI), Scottsdale, AZ 85258, USA; Department of Electrophysiology, HonorHealth, Scottsdale, AZ 85258, USA

**Keywords:** Inappropriate sinus tachycardia, Robotic cardiac surgery, Hybrid ablation

## Abstract

We describe a world-first robotic ablation for inappropriate sinus tachycardia. A 26-year-old woman with refractory inappropriate sinus tachycardia underwent robotic-enhanced hybrid ablation, combining electrophysiological mapping with superior visualization and access compared to video-assisted thoracoscopic surgery (VATS) approaches. Ablations normalized the heart rate from 120 to 70 bpm. At one- and six-month follow-ups, she reported symptom resolution and improved quality of life. Holter monitoring confirmed no tachycardic episodes. This presents a promising alternative for patients who have exhausted conventional treatments, potentially revolutionizing inappropriate sinus tachycardia management.

## INTRODUCTION

Inappropriate sinus tachycardia (IST) presents with unexplained sinus tachycardia (resting >100 bpm, 24 h mean >90 bpm) and symptoms like palpitations, dyspnoea, dizziness and exercise intolerance [1]. Often underdiagnosed, especially in women, IST impairs quality of life and may lead to long-term structural and functional cardiac changes. Effective management is therefore crucial to mitigate adverse outcomes. Conventional treatments (beta-blockers, ivabradine, catheter ablations) often yield suboptimal results with recurrent symptoms, side effects or complications [1]. Robotic-enhanced hybrid ablation (RE-HA) offers a promising, minimally invasive alternative for IST, providing greater access, visualization, precision and safety compared to VATS. This approach may improve outcomes and reduce complications, significantly advancing IST treatment.

## CASE REPORT

A 26-year-old female attended the emergency department with chest pain, leg pain and headaches following occupational chemical exposure, with a year-long history of dizziness and exercise intolerance. Investigations were largely normal; multiple electrocardiograms (EKGs) revealed sinus tachycardia. Diagnosed with IST, she commenced ivabradine and metoprolol therapy, but visual disturbances and dizziness limited her work. Given unsuccessful medical management and the limitations of catheter-based ablations (high recurrence, pacemaker implantation rates) [2] and VATS approaches, RE-HA was chosen.

**Figure 1:**
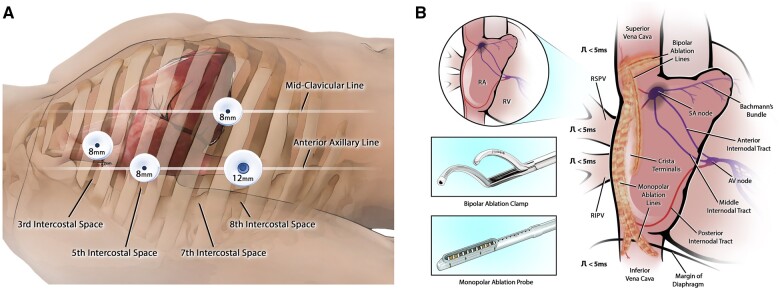
(**A**) Port setup: 8-mm ports were placed in the 3rd, 5th and 7th intercostal spaces with a 12-mm working port in the 8th intercostal space. (**B**) The lesion set was made using the Synergy bipolar ablation clamp and the monopolar EPi-Sense ST ablation probe.

**Figure 2:**
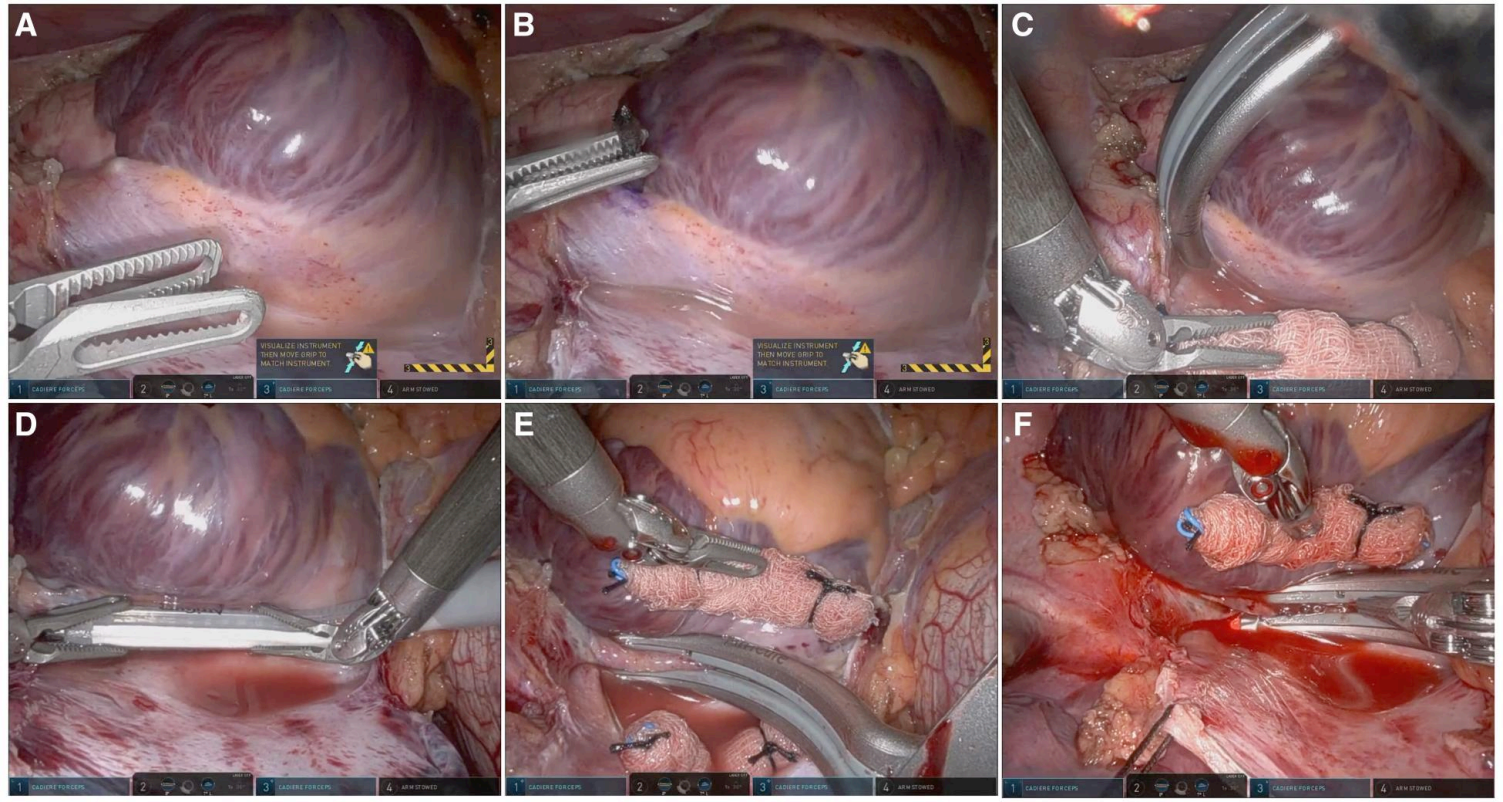
(**A**) Sinoatrial node was identified by mapping the earliest activation site and epicardially via right atrial tenting. (**B**) It was marked with a blue marker. (**C**) The SVC was isolated using the clamp. (**D**) Lesions anchoring the venae cavae were made with the ablation probe. (**E**) Small bites from SVC to mid-RA with the clamp ablated the crista terminalis. (**F**) Two mid-crista lesions were made with the clamp. RA: right atrium; SVC: superior vena cava.

With the patient in the left lateral position, robotic ports were placed (Fig. 1A) for the da Vinci Xi robotic surgical system (Intuitive Surgical, Sunnyvale, CA), and a mapping catheter (Abbott HD Grid, Abbott, Chicago, IL) was placed via the right femoral vein. Using the robotic instruments, the pericardium was opened and endocardial electrophysiological mapping was performed using the mapping catheter, identifying (Fig. 2A) and marking (Fig. 2B) the earliest activation site (the sinoatrial node).

Ablations following the HEAL-IST lesion set (Fig. 1B) [3]. The superior vena cava (SVC) was isolated using the Synergy radiofrequency ablation clamp (Fig. 2C, AtriCure, Mason, OH). Next, the EPi-Sense ST ablation probe (AtriCure, Mason, OH) was used to create 2 sets of lesions (Fig. 2D) anchoring the SVC and inferior vena cava (IVC). Next, the crista terminalis was ablated using the Synergy clamp: 2 lesions from SVC to mid-RA (Fig. 2E), 2 from the lower SVC to the right atrium (RA), and 2 mid-crista lesions (Fig. 2F) taking small bites with the clamp. These were guided by mapping, which identified an active area in the mid-lateral RA wall. Post-ablation remapping was performed to check the lesions’ continuity and transmurality. Six endocardial lesions were made to address active areas identified during remapping, and confirmatory remapping was conducted.

The cardiac surgeon and electrophysiologist collaborated throughout, making real-time decisions based on mapping and ablation response. The initial sinus tachycardia was converted to junctional rhythm (120–70 bpm, 42% reduction). Catheters, sheaths and ports were removed under direct vision, a chest drain was placed in the 7th intercostal space and femoral access sites were closed. Port incisions were closed using subcutaneous 2–0 Stratafix with subcuticular 4–0 Stratafix and Dermabond Prineo (Ethicon, Cincinnati, OH) for skin, with a total procedural time of 2 h 23 min, and ablation time of 53 min. The patient was discharged on the 1st postoperative day in sinus rhythm without complications. At 6-month follow-up, she reported complete symptom resolution with improved quality-of-life. Twenty-four-hour Holter showed mean heart rate of 78 bpm (minimum: 58 bpm during sleep, maximum: 92 bpm) with no sinus tachycardic episodes, supraventricular or ventricular ectopy observed during the recording period.

## DISCUSSION

IST management remains challenging, with conventional treatments often unsuccessful. We present a world-first robotic IST ablation, a significant advancement in treatment. Traditional medical management options can cause side effects (e.g. fatigue, hypotension visual disturbances) as our patient experienced. Catheter ablations, while sometimes beneficial, are technically challenging and risk complications [4]. Hybrid ablation offers a more precise and effective approach than catheter ablation, with RE-HA offering advantages over VATS hybrid approaches, as demonstrated for atrial fibrillation [5].

Superior visualization and access to epicardial structures enables comprehensive ablation, crucial given the sinoatrial node’s complex anatomy. The enhanced dexterity and precision of robotic instruments permit more controlled movements, reducing risk of inadvertent damage. This gentle approach is vital near the sinoatrial node, where preserving functionality while achieving ablation is critical.

This procedure combined endocardial mapping with epicardial and endocardial ablations, tailored to patient-specific anatomy and electrophysiology. The lesions targeting the SVC, IVC, crista and RA achieved significant heart rate reduction without permanent pacing. Using the ablation clamp and probe, comprehensive linear ablation lesions were created, effectively isolating the RA and targeting potential sources of abnormal electrical activity. This approach improves upon conventional catheter approaches, which often result in high recurrence rates or require pacemaker implantation.

While more invasive than percutaneous approaches, the potential benefits in efficacy and durability may outweigh this drawback for patients who have exhausted other options. Rapid recovery and significant symptom improvement at 1 month are encouraging, long-term data are crucial to evaluate durability. Key questions include potential long-term recurrence, impact on cardiac structure and function and the learning curve. While the superiority of this approach cannot be concluded from a single case, larger studies with extended follow-up will evaluate long-term efficacy, compare outcomes with conventional treatments to influence clinical practice and assess the generalizability of our results. While promising, pulsed field ablation was not considered due to limited evidence and safety concerns at present. Future studies should compare hybrid and RE-HA approaches with such emerging technologies.

We present a promising alternative for refractory IST, with potentially more effective and durable outcomes. With refinement and long-term data, RE-HA could be a valuable addition to the armamentarium for treating IST.

## Data Availability

The original patient data are confidential and therefore cannot be shared. Anonymized data may be available upon request to the corresponding author.
